# Development and validation of a MRI-based combined radiomics nomogram for differentiation in chondrosarcoma

**DOI:** 10.3389/fonc.2023.1090229

**Published:** 2023-02-28

**Authors:** Xiaofen Li, Min Lan, Xiaolian Wang, Jingkun Zhang, Lianggeng Gong, Fengxiang Liao, Huashan Lin, Shixiang Dai, Bing Fan, Wentao Dong

**Affiliations:** ^1^ Medical College of Nanchang University, Nanchang, China; ^2^ Department of Radiology, Jiangxi Provincial People’s Hospital, The First Affiliated Hospital of Nanchang Medical College, Nanchang, China; ^3^ Department of Orthopedics, Jiangxi Provincial People’s Hospital, The First Affiliated Hospital of Nanchang Medical College, Nanchang, China; ^4^ Department of Radiology, The Affiliated Hospital of Jiangxi University of Traditional Chinese Medicine, Nanchang, China; ^5^ Department of Medical Imaging Center, Second Affiliated Hospital of Nanchang University, Nanchang, China; ^6^ Department of Nuclear Medicine, Jiangxi Provincial People’s Hospital, The First Affiliated Hospital of Nanchang Medical College, Nanchang, China; ^7^ Department of Pharmaceutical Diagnosis, General Electric Healthcare, Changsha, China

**Keywords:** chondrosarcoma, radiomics, nomogram, magnetic resonance imaging, T1-weighted image, prediction tool

## Abstract

**Objective:**

This study aims to develop and validate the performance of an unenhanced magnetic resonance imaging (MRI)-based combined radiomics nomogram for discrimination between low-grade and high-grade in chondrosarcoma.

**Methods:**

A total of 102 patients with 44 in low-grade and 58 in high-grade chondrosarcoma were enrolled and divided into training set (n=72) and validation set (n=30) with a 7:3 ratio in this retrospective study. The demographics and unenhanced MRI imaging characteristics of the patients were evaluated to develop a clinic-radiological factors model. Radiomics features were extracted from T1-weighted (T1WI) images to construct radiomics signature and calculate radiomics score (Rad-score). According to multivariate logistic regression analysis, a combined radiomics nomogram based on MRI was constructed by integrating radiomics signature and independent clinic-radiological features. The performance of the combined radiomics nomogram was evaluated in terms of calibration, discrimination, and clinical usefulness.

**Results:**

Using multivariate logistic regression analysis, only one clinic-radiological feature (marrow edema OR=0.29, 95% CI=0.11-0.76, P=0.012) was found to be independent predictors of differentiation in chondrosarcoma. Combined with the above clinic-radiological predictor and the radiomics signature constructed by LASSO [least absolute shrinkage and selection operator], a combined radiomics nomogram based on MRI was constructed, and its predictive performance was better than that of clinic-radiological factors model and radiomics signature, with the AUC [area under the curve] of the training set and the validation set were 0.78 (95%CI =0.67-0.89) and 0.77 (95%CI =0.59-0.94), respectively. DCA [decision curve analysis] showed that combined radiomics nomogram has potential clinical application value.

**Conclusion:**

The MRI-based combined radiomics nomogram is a noninvasive preoperative prediction tool that combines clinic-radiological feature and radiomics signature and shows good predictive effect in distinguishing low-grade and high-grade bone chondrosarcoma, which may help clinicians to make accurate treatment plans.

## Introduction

Chondrosarcoma is a malignant bone tumor characterized by the production of cartilage matrix, which accounts for about 20% of all primary malignant bone tumors (1). After myeloma and osteosarcoma, chondrosarcoma is the third most common primary bone malignant tumor. Chondrosarcoma occurs in a wide range of ages, more males than females, mostly in long tubular bones, mostly in the proximal femur and humerus, and rarely in the intracranial, larynx, sacrum, and sternum. According to the 5th edition of WHO Classification of Bone Neoplasms in 2020, conventional chondrosarcoma is classified into grade I, grade II, and grade III (2, 3). Different grades of chondrosarcoma have widely different treatment methods and prognosis (4). The main clinical treatment is tumor resection to prevent recurrence and distant metastasis (5). Studies have confirmed that low-grade chondrosarcoma does not metastasize, and the 10-year survival rate of grade I chondrosarcoma is about 77-89% (6). Therefore, curettage of the lesion is safe and effective. About 10-33% of grade II chondrosarcomas and 70% of grade III chondrosarcomas can metastasize, with 10-year survival rates of about 53-59% and 36-38%, respectively, requiring extensive resection or amputation (7). Due to the heterogeneity of the tumor and biopsy of small sampling, and the gross results have a certain difference, can not accurately predict the tumor differentiation degree, affect the clinical risk of illness misjudgment leads to increased postoperative recurrence or metastasis, therefore, noninvasive accurate preoperative assessment of the precision medical treatment and prognosis in patients with chondrosarcoma is particularly important.

On conventional X-ray and CT, puncture, annular, semi-annular, or gravel calcification were seen in the solid portion of the chondrosarcoma, and scallop-like changes were seen in the adjacent cortical bone (8). Due to its high soft tissue resolution, MRI has been widely used in the diagnosis and differential diagnosis of chondrosarcoma, especially for the morphological characteristics of the tumor, such as marrow edema, soft tissue mass, periosteal reaction, etc. (9). However, these features extracted from traditional imaging have certain limitations in predicting the differentiation degree of chondrosarcoma (10).

In recent years, radiomics through different kinds of machine algorithm to extract the quantitative features of high flux, and mining related to tumor heterogeneity, or the degree of differentiation of biological information, has been widely applied in glioma, the preoperative differentiation degree of prediction and prognosis of gastric cancer, colorectal cancer evaluation (11–14). Meanwhile, some researches focused on identification of chondrosarcoma and enchondroma radiomics study (15). The objective of our study was to develop and validate a combined radiomics nomogram that combines unenhanced MRI radiomic features with clinic-radiological features for preoperative differentiation of low-grade (grade I) and high-grade (grade II, III) chondrosarcomas.

## Materials and methods

### Patients

Institutional Review Board approval and a waiver for informed consent were obtained. This retrospective research included patients with low-to-high grade chondrosarcoma who underwent MRI, between June 2015 and January 2022, at our Hospital. The inclusion criteria were as follows: (1) primary low-grade and high-grade chondrosarcoma that underwent surgery, such as intralesional curettage or resection. (2) the definitive histological diagnosis was based on the evaluation of the surgical specimen and was regarded as a reference standard. (3) MRI was performed within three months before surgery and T1WI and T2WI sequences were provided. The exclusion criteria were as follows: (1) patients received radiotherapy or chemotherapy before MRI scans. (2) poor imaging quality making difficulties in segmentation. Finally, a total of 102 patients with low-grade (n=44, 17 male and 27 female) and high-grade (n=58, 31 male and 27 female) chondrosarcoma were included in the present study.

44 low-grade chondrosarcoma patients. These lesions were located in the rib (n = 5), sternum (n = 2), pelvis (n = 4), humerus (n=8), tibia (n=8), femur (n=10), radius (n=4) and scapula (n = 3), respectively.

58 high-grade chondrosarcoma patients. These lesions were located in the sternum (n=2), pelvis (n=16), humerus (n=15), tibia (n=3), femur (n=16), scapula (n = 3), calcaneus (n=1), sacrum (n=1) and patella (n=1), respectively.

Patients were randomly assigned to the training and validation sets in a ratio of approximately 7:3. The training and validation sets were stratified to maintain the same proportion of low-grade and high-grade tumors in the training and validation groups.

### MRI image acquisition

All enrolled patients underwent 3.0T MRI (SIMENS MAGNETOM Skyra) with at least T1WI and T2WI MRI sequences. T1WI scanning parameters: TR619ms, TE12ms, F0V390mm, matrix 384×384, layer thickness 5mm, interval 1mm, reverse Angle 160°, echo chain length 3. T2WI scanning parameters: TR3400ms, TE85ms, FOV380mm, matrix 384×384, layer thickness 5mm, interval 1mm, reverse Angle 160°, echo chain length 15.

### MRI characteristic evaluation

The MRI image were scrutinized by two radiologists with 5 years (doctor 1, D.W.) and 15 years (doctor 2, L.X.) of diagnostic bone imaging experience. Blinded to the clinic-pathologic data, the two doctors interpreted the following MRI features by consensus: “Longest diameter” were the longest diameter of the tumor on an axial MRI image; Periosteal reaction (present or not; “periosteal reaction” were fluid signal intensity on T2W sequences on the surface of the bone); Marrow edema (present or not; “marrow edema” were patch of hyperintensity around the tumor on the T2W sequences); Soft tissue mass: (present or not; “soft tissue mass” were intramedullary cartilage tumors extend directly through the damaged cortex and have the same radiographic features and well-defined peripheral margins as intramedullary tumors). If there was disagreement, the two radiologists needed to reach a consensus.

### Construction of the clinic-radiological model

The clinical parameters of gender and age were retrieved from the electronic medical record system of our hospital. Two radiologists with 5 and 15 years of experience in bone imaging were blinded to imaging reports and pathological details, and imaging features including longest diameter, periosteal reaction, marrow edema and soft tissue mass were reviewed and reported. When constructing the clinic-radiological model, the above clinic-radiological characteristics were analyzed by univariate regression, and then the statistically significant characteristics in the univariate regression analysis were processed by multivariate regression model. Ultimately, features with P values less than 0.05 were used to establish the clinic-radiological model.

### Tumor segmentation

**Figure 1:**
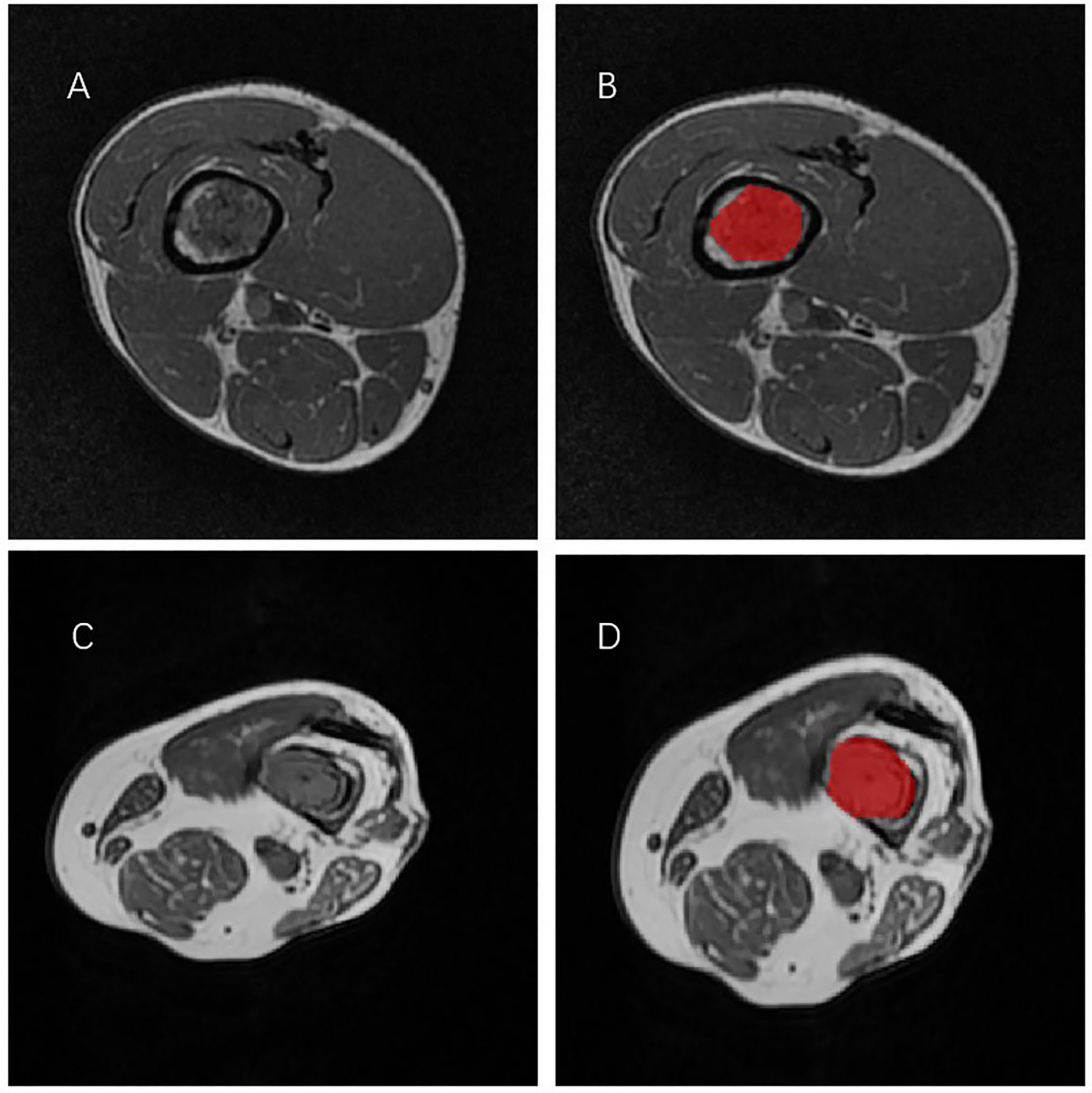
Two representative T1WI MRI segmentation on ITK-SNAP. **(A, B)** A patient with low-grade chondrosarcoma of the tibia. **(C, D)** A patient with high-grade chondrosarcoma of the tibia.

All T1-weighted MRI images in DICOM format, original size and resolution were transferred to ITK-SNAP software (Version 3.8, www.itksnap.org) for three-dimensional (3D) region of interest (ROI) segmentation. To ensure accurate tumor boundaries, ROIs on all slices were carefully delineated manually by a radiologist (doctor 1, D.W.) with 5 years of experience in bone imaging, who was blinded to the pathological findings. To test the stability of features, doctor 1 and another radiologist with 15 years of experience (doctor 2, L.X.) underwent re-extraction of radiomic features from 40 randomly selected patients from the entire study set. Intra-class correlation coefficient (ICC) was calculated to evaluate the consistency and reproducibility of the features. Subsequent analyses included features of ICC>0.75 in intra-observer and inter-observer consistency analyses. In order to avoid local volume effect, the top layer and bottom layer were eliminated. **Figure 1** shows two typical T1WI MRI images of chondrosarcoma segmentation on ITK-SNAP, one of which is low-grade (**Figures 1A, B**) and the other of which is high-grade (**Figures 1C, D**).

### Radiomics feature extraction

Prior to radiomics feature extraction, to reduce feature variability, we performed the following image preprocessing steps, containing gray discretization, intensity normalization and voxel resampling. Then, radiomics features were extracted from T1WI MRI images through the open source PyRadiomics library. They are divided into four categories: size and morphology features, descriptors of image intensity histograms, descriptors of the relationship between image voxels and higher-order texture features extracted from filtered images.

### Construction of the radiomics signature

In order to prevent signature overfitting, dimensionality of features is reduced before signature construction. Succinctly, radiomics features that met the inter-observer and intra-observer ICCs criteria greater than 0.75 and were significantly different between the two groups as assessed by one-way analysis of variance (ANOVA) were included in the LASSO regression model to select the most valuable features in the training set. Finally, the selected radiomics features were used to construct radiomics signature. Rad-score was calculated for each patient by linear combination of selected features and weighted by the respective LASSO coefficients.

### Development and validate of combined radiomics nomogram

A combined radiomics nomogram was developed by combining significant variables of clinic-radiological characteristics and Rad-score. The calibration curve was used to evaluate the calibration of nomogram. Hosmer-Lemeshow test evaluated the goodness of fit of nomogram. The ROC curve of the training and validation sets and DeLong tests were used to evaluate the diagnostic performance of the clinic-radiological model, radiomics signature and combined radiomics nomogram in differentiating low-grade from high-grade in chondrosarcoma. DCA was performed by calculating the net benefits over a range of threshold probabilities across the entire set, in order to assess the clinical usefulness of the nomogram.

### Statistical analysis

Statistical analysis was conducted using SPSS 19.0 and R software (Version 3.4.4; http://www.Rproject.org). The chi-square test was used for the analysis of categorical variables and the Mann-Whitney U test was used for the analysis of continuous variables. The clinic-radiological factor and Rad-score were correlated using Pearson’s correlation coefficient. The “glmnet (R)” package was used to perform LASSO regression. The “Regression Modeling Strategy (RMS)” package was used to construct the nomogram and calibration curves. The Hosmer–Lemeshow test was performed on the”generalhoslem” package. ROC curves were plotted using the “partial Receiver Operating Characteristic (pROC)” software package. Delong test was used to estimate the difference of AUC values between different models (clinic-radiological model, radiomics signature, combined radiomics nomogram). Risk Model Decision Analysis (RMDA) software package was used to analyze DCA. The significance level was set as two-sided p < 0.05.

## Results

### Clinic-radiological characteristics and construction of the clinic-radiological model

A total of 102 patients were enrolled in our study, and clinical and radiological data were collected. **Table 1** summarizes the differences in clinical radiological variables between low-grade and high-grade chondrosarcoma patients in the training and validation sets. The training set including 72 patients (36 males and 36 females), of whom 31 were diagnosed with low-grade chondrosarcoma and 41 with high-grade chondrosarcoma. Patients with high-grade chondrosarcoma was only significantly different from those with low-grade chondrosarcoma in terms of marrow edema (p=0.02 in the training set). Univariate analysis showed that marrow edema and periosteal reaction served as the risk factors of low-grade and high-grade in chondrosarcoma. After multivariate logistic regression analysis, only marrow edema (OR=0.29, 95% CI=0.11-0.76, p=0.012) remained an independent clinic-radiological predictor (**Table 2**).

**Table 1:** Clinic-radiological characteristics of chondrosarcoma patients in the training and validation sets.

characteristics	Training set (n=72)		p-value	Validation set (n=30)		p-value
	Low grade (n=31)	High grade (n=41)		Low grade (n=13)	High grade (n=17)	
Gender, n (%)						
Male	14(45.2)	22 (53.7)		3 (23.1)	9 (52.9)	
Female	17(54.8)	19(46.3)	0.634	10 (76.9)	8 (47.1)	0.201
Age(Y)	45.2 ± 14.6	46.2 ± 17.9	0.795	49.8 ± 18.5	51.5 ± 16.9	0.795
Longest diameter(cm)	5.1 ± 3.2	4.3 ± 2.7	0.262	6.5 ± 3.6	4.2 ± 1.8	0.022
Periosteal reaction, n (%)	14 (45.2)	9 (22.0)	0.066	7 (53.8)	7 (41.2)	0.749
Marrow edema, n (%)	20 (64.5)	14 (34.1)	0.020	8 (61.5)	9 (52.9)	0.921
Soft tissue mass, n (%)	17 (54.8)	15 (36.6)	0.192	9 (69.2)	8 (47.1)	0.399

**Table 2:** Univariate and multivariate logistic regression analysis of the clinic-radiological features in predicting the high-grade and low-grade chondrosarcoma.

Variable	Univariate regression		Multivariate regression	
	OR (95%CI)	P-value	OR (95%CI)	P-value
Gender	1.406[0.551-3.587]	0.476	NA	NA
Age	1.004[0.976-1.032]	0.792	NA	NA
Longest diameter	0.913[0.778-1.071]	0.264	NA	NA
Periosteal reaction	0.342[0.123-0.950]	0.0396	NA	NA
Marrow edema	0.285[0.107-0.759]	0.0119	0.29[0.11-0.76]	0.012
Soft tissue mass	0.475[0.184-1.229]	0.125	NA	NA

### Feature extraction, selection, and radiomics signature building

**Figure 2:**
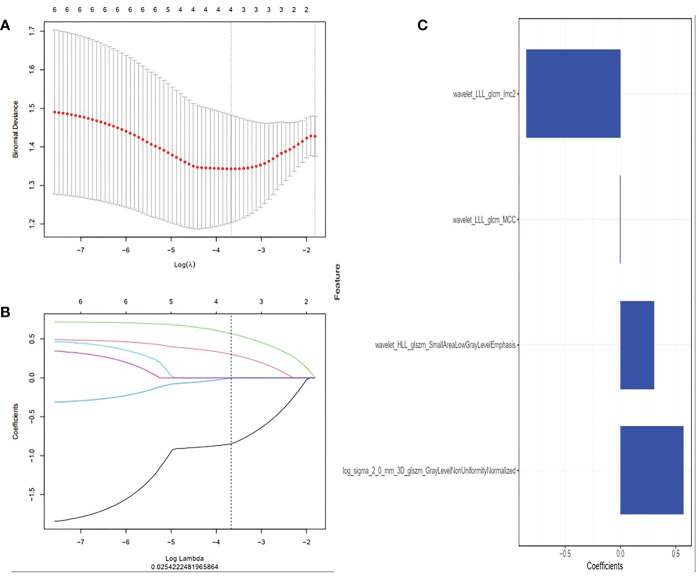
Radiomics features were selected using the least absolute shrinkage and selection operator (LASSO) regression. **(A)** Selection of tuning parameter (λ) in LASSO model. Selected the optimal value with λ= 0.025 and log(λ) =-3.605. **(B)** LASSO coefficient profiles of radiomics features. The coefficient profiles corresponding to the selected logarithm (λ) values were generated by five-fold cross validation. **(C)** Selected radiomics features and their coefficients.

A total of 1316 radiomics features were extracted from T1WI MRI images of each chondrosarcoma patient, among which 829 features were proved to have good inter-observer and intra-observer agreement, which ICCs achieve greater than 0.75. The significant difference between high-grade and low-grade chondrosarcoma in one-way ANOVA (P < 0.05) was enrolled into the LASSO logistic regression model to select the most valuable radiomics features (**Figures 2A, B**). Finally, four radiomics features were used to construct radiomics signature (**Figure 2C**).

### Development and validate of combined radiomics nomogram

**Figure 3:**
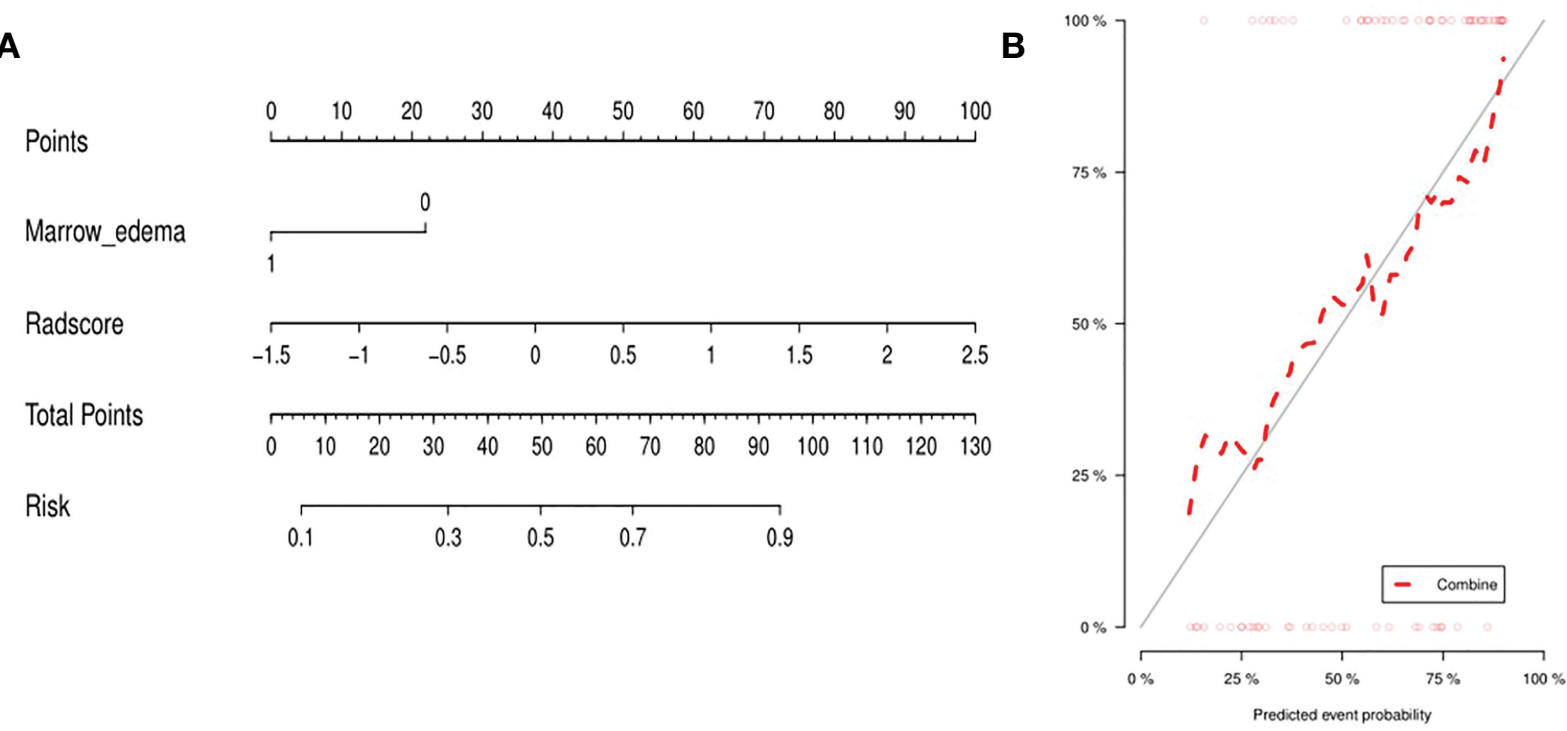
**(A)** Integrating marrow edema and radiomics signature, the MRI-based nomogram was established. **(B)** For the calibration curve of the nomogram, the closer the fit between the red diagonal and the ideal dashed line, the higher the prediction accuracy of the nomogram.

The clinic-radiological factor (marrow edema) and Rad-score were incorporated into construction of the MRI-based combined radiomics nomogram (**Figure 3A**). After spearman correlation analysis, the result was that R values were all very small, and P values were all > 0.05, which indicated that there was no correlation between marrow edema and Rad-score, and they were used to construct the combined radiomics nomogram from different perspectives. The calibration curve (**Figure 3B**) showed a good agreement between the predicted and actual probabilities for predicting the low-grade and high-grade chondrosarcoma in the training and validation sets, and the Hosmer-Lemeshow test yielded a nonsignificant statistical difference (P =0.169 and 0.317).

**Figure 4:**
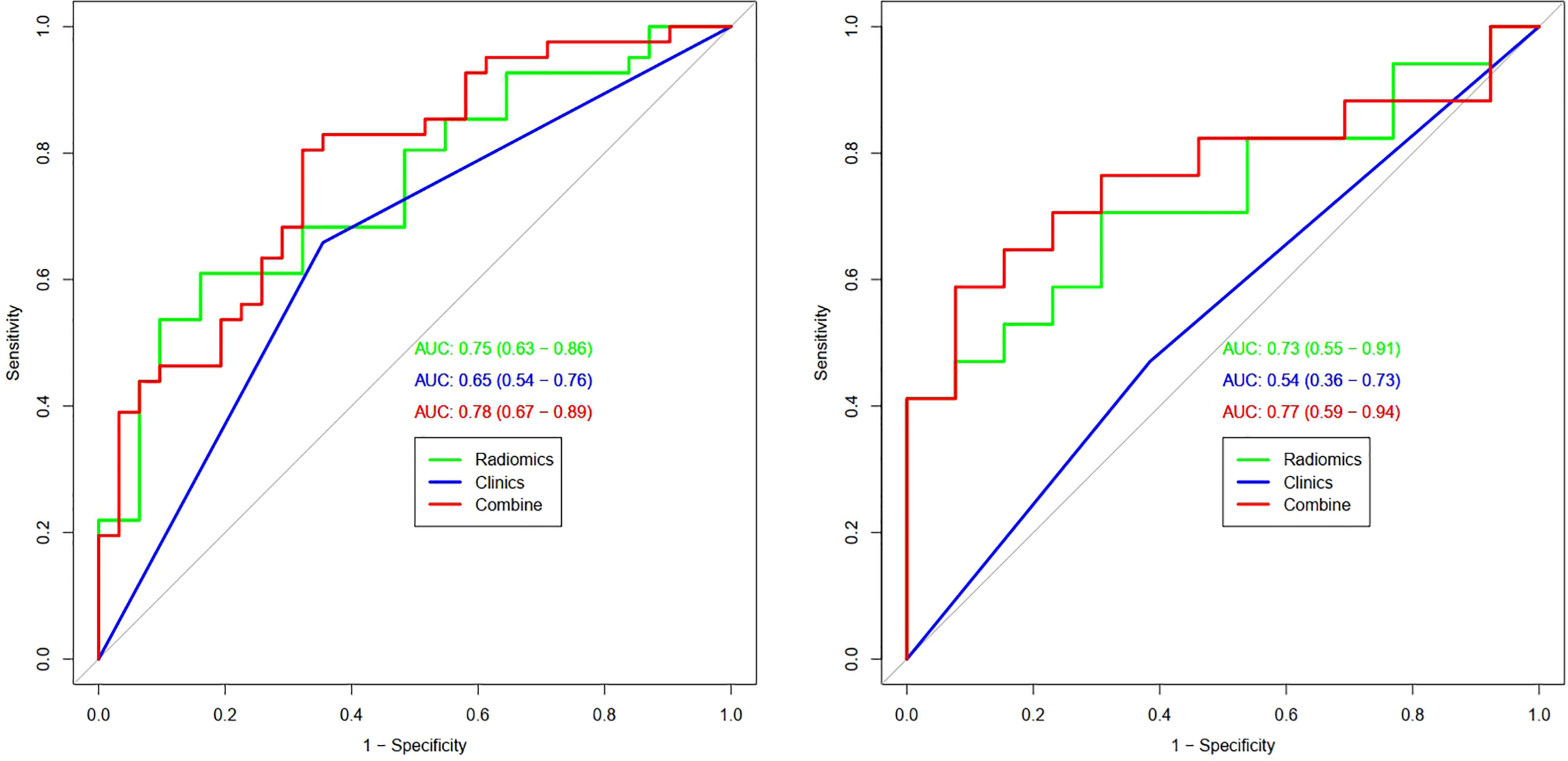
ROC curves (AUC) of the three models in the training (left) and validation sets (right).

**Figure 5:**
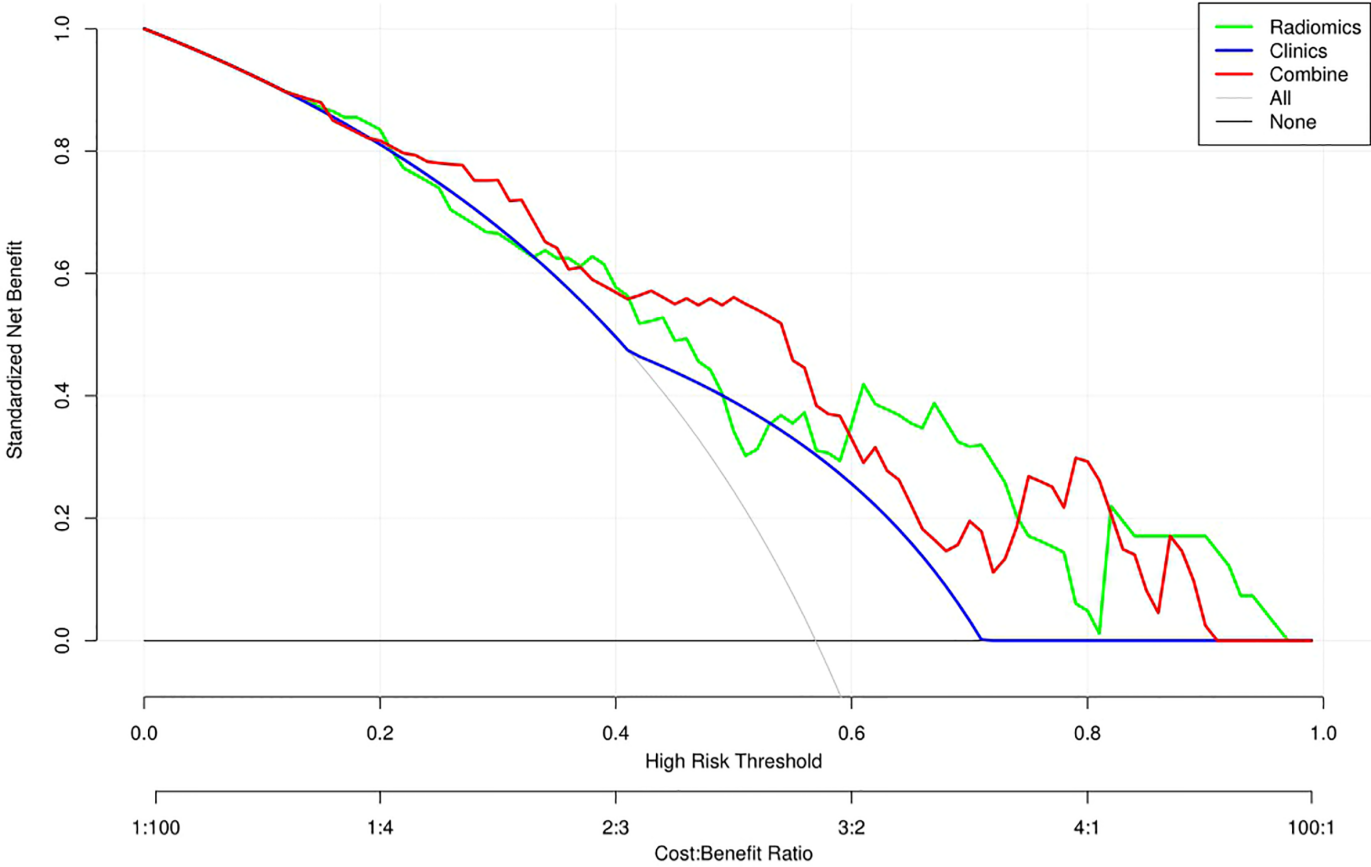
Decision curve analysis (DCA) for the three models. The DCA showed that more net benefits were obtained using the combined radiomics nomogram approach over most threshold probability ranges.

**Table 3** and **Figure 4** present the clinic-radiological, radiomics signature and combined radiomics nomogram diagnostic performance and ROC curves in the training and validation sets, respectively. The AUC of the clinic-radiological model was 0.65 (95%CI 0.54-0.76) in the training set and 0.54 (95%CI 0.36-0.73) in the validation set, while the radiomics signature yielded an AUC value of 0.75 (95%CI 0.63-0.86) and 0.73 (95%CI 0.55-0.91) in both sets. The combined radiomics nomogram achieved the optimal discrimination in the training (AUC, 0.78; 95% CI, 0.67-0.89) and validation (AUC, 0.77; 95% CI, 0.59-0.94) sets, with accuracy of 0.750 and 0.700, sensitivity of 0.805 and 0.765, and specificity of 0.677 and 0.615, respectively. Using the Delong test (**Table 4**), there was a significant difference between the clinic-radiological and the MRI-based combined radiomics nomogram AUC in the training set (P = 0.013) and the validation set (P =0.037), but the difference did not reach statistical significance between nomogram and radiomics (P=0.337, 0.491) and between clinic-radiological and radiomics (P=0.230, 0.208), respectively, in the training set and validation set. The DCA was shown in **Figure 5**. The combined radiomics nomogram demonstrated a higher overall net benefit than radiomics model, indicating that the combined radiomics nomogram had an excellent clinical application value in distinguishing high-grade from low-grade chondrosarcoma.

**Table 3:** Predictive performance of clinic-radiological model, radiomics signature, and combined radiomics nomogram.

Model	Radiomics		Clinics		Combine	
	Training	Validation	Training	Validation	Training	Validation
Accuracy(95%CI)	0.708 (0.589-0.810)	0.667 (0.472-0.823)	0.653 (0.531-0.761)	0.533 (0.343-0.717)	0.750 (0.634-0.845)	0.700 (0.506-0.853)
Sensitivity (%)	0.610	0.588	0.695	0.471	0.805	0.765
Specificity (%)	0.839	0.769	0.645	0.615	0.677	0.615
Pos.Pred.Value	0.833	0.769	0.711	0.615	0.767	0.722
Neg.Pred.Value	0.619	0.588	0.588	0.471	0.724	0.667

**Table 4:** Comparison of the prediction with the combined radiomics nomogram, clinic-radiological model, and radiomics signature.

Group	Model1	Model2	P-value
Training	Nomogram	Clinics	0.013
	Nomogram	Radiomics	0.337
	Clinics	Radiomics	0.230
Validation	Nomogram	Clinics	0.037
	Nomogram	Radiomics	0.491
	Clinics	Radiomics	0.208

## Discussion

In this retrospective study, we developed and validated a combined radiomics nomogram for noninvasive, individualized prediction of low-grade and high-grade chondrosarcoma. The combination of clinic-radiological features and T1WI MRI radiomics signature showed predictive efficiency (AUC=0.77, 95%CI =0.59-0.94) in distinguishing chondrosarcoma patients with satisfactory reproducibility and reliability.

Accurate grading of chondrosarcoma is an urgent need to select the most appropriate treatment. For low-grade lesions, conservative treatment is recommended, and for high-grade lesions, aggressive treatment is required (4, 16). However, preoperative biopsies may mistakenly lead to a reduction in chondrosarcoma grade because only small lesion areas are sampled, interobserver differences in tumor grade can occur even among professional bone pathologists (17, 18). Furthermore, inaccurate preoperative grading may lead to inadequate treatment, subsequent need for further surgery, and increased morbidity.

Before surgery, imaging plays a crucial role, and MRI is the preferred method (9). H. Douis et al. found that periosteal reaction and soft tissue mass on MRI may indicate a high degree of malignancy in patients with chondrosarcoma (19). Our qualitative image analysis was less consistent with this finding, only marrow edema was proved to be independent factors of high-grade chondrosarcomas (OR=0.29, 95% CI=0.11-0.76, P=0.012), probably due to the sample of his research and ours were not large and statistical bias may occur.

We only extract tumor texture features on the T1WI sequence, because the boundary of the tumor is easy to delineate on the T1WI sequence. T1WI sequence which is the most generalized and has the most stable image quality was adopted as the research sequence, and all T1WI images were from the same device. We only used T1WI sequence to have a radiomics model, which had a similar diagnostic efficacy, indicating that our study was very effective. In addition, T2WI sequence and diffusion-weighted MRI have been demonstrated unable to distinguish low-grade from high-grade chondrosarcomas (10, 20). Besides, contrast-enhanced MRI (CE-MRI) is helpful in diagnosis because of the rapid enhancement of high-grade tumor areas due to rich vascularized intralesional septations (21, 22). However, CE-MRI were not evaluated in our research because not all patients underwent CE-MRI scans.

At present, imaging techniques could be further improved to better grade and accurately diagnose chondrosarcoma before surgery (23). With the development of artificial intelligence, radiomics can provide valuable assistance by providing quantitative data to integrate existing qualitative image information (24). Recently, the radiomics studies of chondrosarcoma mainly focus on the differentiation of enchondroma from chondrosarcoma, chondrosarcoma recurrence or survival evaluation, limited studies have focused on radiomics and chondrosarcoma differentiation and grading. To our knowledge, Gitto S and his team did some study on chondrosarcoma differentiation (10), they evaluated 58 patients with low-grade and high-grade chondrosarcoma and observed that MRI-based volumetric texture analysis could distinguish these two lesions by many separate texture features. Recently, they also investigated the diagnostic performance of machine learning model based on MRI-based radiomics in distinguishing atypical cartilaginous tumour (ACT) from grade II chondrosarcoma in long bones (25). However, these studies established a machine learning model based only on radiomics features, ignoring the importance of clinical and radiological features. For computerized clinical decision support systems, radiomics-derived data are not a panacea. Our research summarizes the subtle differences between clinical radiologic features and MRI radiomics features in patients with chondrosarcoma and analyses them with nomogram. We chose to investigate several indicators in terms of clinical information that could independently predict the grade of differentiation.

Limitations of our study deserve consideration. First, this is a retrospective study, the performance of the training model is limited by the sample size and only comes from single-center tertiary hospitals, which lacks external validation. It is necessary to carry out large-sample and multicenter studies in the future, and the efficiency of the model should be further tested. Second, more clinical factors and MRI sequence need to incorporated into our nomogram, especially contrast-enhanced T1WI sequence. Future studies will focus on improving the predictive power by combining multiparametric MRI data with other clinical factors and validating the predictive nomogram on multicenter data.

In conclusion, our findings suggest that combined radiomics nomogram based on T1WI sequential MRI have great potential as biomarkers for differentiating low-grade from high-grade chondrosarcomas. As a non-invasive, preoperative method, the combined radiomics nomogram may helpful for the differentiation and clinical decision-making of chondrosarcoma patients.

## Data availability statement

The original contributions presented in the study are included in the article/supplementary material. Further inquiries can be directed to the corresponding authors.

## Ethics statement

The studies involving human participants were reviewed and approved by the ethics committee of the Jiangxi Provincial People’s Hospital. Written informed consent for participation was not required for this study in accordance with the national legislation and the institutional requirements.

## Author contributions

The authors made the following contributions: WD and XL made the conception for this research. Data collection and analysis were performed by WD, ML, XW, JZ, FL and SD. WD, ML and XW analyzed the data and drafted the article. XL, BF, LG and HL reviewed/edited the manuscript. All the authors critically revised the article for important intellectual content. All authors contributed to the article and approved the submitted version.
